# Silver-loaded nanoparticles affect *ex-vivo* mechanical behavior and mineralization of dentin

**DOI:** 10.4317/medoral.22885

**Published:** 2019-03

**Authors:** Manuel Toledano, Fátima S. Aguilera, Inmaculada Cabello, Manuel Toledano-Osorio, Estrella Osorio, Modesto T. López-López, Franklin García-Godoy, Franklin Lynch, Raquel Osorio

**Affiliations:** 1 DDS, PhD, Professor. Dental Materials Section, University of Granada, Faculty of Dentistry, Colegio Máximo de Cartuja s/n, Granada, Spain; 2 DDS, PhD, Research Fellow. Dental Materials Section, University of Granada, Faculty of Dentistry, Colegio Máximo de Cartuja s/n, Granada, Spain; 3 Research Fellow. Dental Materials Section, University of Granada, Faculty of Dentistry, Colegio Máximo de Cartuja s/n, Granada, Spain; 4 DDS, PhD, Professor. University of Granada, Faculty of Science, Applied Physics Department. Fuente Nueva s/n 18071 – Granada - Spain; 5 DDS, MS, PhD, Professor. Bioscience Research Center, College of Dentistry, University of Tennessee, Health Science Center, 875 Union Avenue, USA; 6 BDS, PhD, Professor. University Dental School & Hospital/ University College Cork, Wilton, Cork – Ireland

## Abstract

**Background:**

The aim was to evaluate the effect of silver loaded nanoparticles (NPs) application on the triboscopic, crystallographic and viscoelastic properties of demineralized dentin. Polymethylmetacrylate-based NPs and Ag loaded NPs were applied on demineralized dentin.

**Material and Methods:**

Treated and untreated surfaces were probed by a nanoindenter to test viscoelasticity, and by atomic force microscopy to test nanoroughness and collagen fibril diameter. X-ray diffraction and transmission electron microscopy through selected area diffraction and bright-field imaging were also used.

**Results:**

Dentin treated with Ag-NPs attained the lowest complex modulus, and the highest tan delta values after 7 days of storage. Dentin treated with undoped-NPs achieved the lowest nanoroughness and the greatest collagen bandwidths among groups. Crystals were identified as hydroxyapatite with the highest crystallographic maturity and crystallite size in dentin treated with undoped-NPs. Texture increased in all samples from 24 h to 7 d, except in dentin surfaces treated with Ag-NPs at 310 plane. Polyhedral, block-like, hexagonal or plate-like shaped apatite crystals constituted the bulk of minerals in dentin treated with Ag-NPs, after 7 d. Polyhedral or rounded/drop-like, and polymorphic in strata crystal apatite characterized the minerals when undoped-NPs were used, with more crystalline characteristics after 7 d than that found when Ag-NPs were applied. Ag-NPs application did not improve the mechanical performance of dentin and did not produce dentin remineralization. However, energy was dissipated through the dentin without showing stress concentration; contrary was occurring at dentin treated with undoped-NPs, that provoked bridge-like mineral deposits at the dentin surface.

**Conclusions:**

Ag-NPs application did not enhance the mechanical properties of cervical dentin, though the energy dissipation did not damage the dentin structure. Remineralization at dentin was not produced after Ag-NPs application, though improved crystallinity may lead to increase stability of the apatite that was generated at the dentin surface.

** Key words:**Dentin, mechanical, mineralization, roughness, silver, viscoelastic.

## Introduction

To facilitate dentin remineralization and antimicrobial characteristics, dentin infiltration with cytocompatible polymeric nanoparticles (NPs), such as calcium and phosphate sequestering materials have been previously proposed (1-3). Silver has been included into some dentin adhesive formulations (3) as it has demonstrated to be an effective antimicrobial, able to promote caries arrestment and to prevent biofilm formation (4). NPs-loaded with Ag play an important role in the disinfection of dentinal tubules (5).

In most of the previous studies, there is a lack of information dealing with the beneficial or harmful effects of silver on hard tissues regeneration with apparent inconsistencies in the results obtained. Silver not only provides an antibacterial effect, but biochemical inertness (6). To date, only a few studies have focused on the effect of silver on bone regeneration associated with implanted materials (7). Nevertheless, recently, Dong *et al.*, 2017 (8) described a bridge formed with new bone occupying a big area of a cranial created defect, after a gelatin-Ag application (8).

Ag-NPs regulate the deposition of collagen and inhibit uncontrolled growth of collagen, as well as directing proper collagen matrix alignment and spatial arrangement (9). Although there are few studies of Zn or Ca-loaded NPs (1,2) on dentin, the effects of Ag-NPs on the dynamic mechanical properties and physic-chemical characterization of treated dentin are unknown.

Nano-dynamic mechanical analysis (Nano-DMA), atomic force microscopy (AFM) analysis, X-ray diffraction (XRD), transmission electron microscopy (TEM) and selected area electron diffraction (SAED) were combined to examine the new formed dentin under the effects of silver. Therefore, the purpose of this study was to assess the effect of Ag-NPs on the triboscopic properties, and both crystallographic and viscoelastic performance of cervical dentin. The null hypothesis that was established is that Ag is not able to create differences in dynamic mechanical performance, triboscopic properties and crystal morphology of dentin.

## Material and Methods

-Nanoparticles production and specimens’ preparation

PolymP-n Active nanoparticles (NPs) (NanoMyP, Granada, Spain) were fabricated through polymerization precipitation (10). NPs are composed by 2-hydroxyethyl methacrylate (backbone monomer), ethylene glycol dimethacrylate (cross-linker) and methacrylic acid (functional monomer). For silver complexation, 30 mg of NPs were immersed at room temperature, during 3 days under continuous shaking in 15 ml aqueous solution of AgNO3 (containing Ag at 40 ppm at pH 6.5), in order to reach the adsorption equilibrium of metal ions. Two different NPs were produced: 1) undoped-NPs (NPs), 2) NPs doped with Ag (Ag-NPs). Nine sound, human single-rooted teeth were obtained with informed consent from donors (18 to 25 yr of age), under a protocol approved by the Institution review board (#405/CEIH/2017). From each tooth, two dentin blocks were obtained from the buccal surface of the root, just below the cementodentinal junction. The tooth was cut perpendicular to the axial axis using a diamond saw (Accutom-50 Struers, Copenhagen, Denmark) under copious water irrigation to obtain cementodentinal slabs with a thickness of 1 mm. Specimens were then prepared as in Toledano-Osorio *et al.*, 2018 (11). A phosphate buffered suspension (PBS) of NPs (10 mg/ml), Ag-NPs (10 mg/ml) or just a PBS solution were applied (30 s), in each of the three different experimental groups. Half of the specimens were tested after storing in PBS at 37º C for 24 hours and the other half after 7 days.

-Nano-DMA and Atomic Force Microscopy (AFM) analysis

Blocks of each treated dentin were subjected to nano-DMA and AFM analyses. Nanomechanical properties were assessed by means of a Hysitron Ti Premier nanoindenter (Hysitron, Inc., Minneapolis, MN), a commercial nano-DMA package. A dynamic (oscillatory) force of 2 µN was superimposed on the quasistatic signal at a frequency of 200 Hz. Modulus mapping of our samples was conducted by imposing a quasistatic force setpoint, Fq=2 µN, to which we superimposed a sinusoidal force of amplitude FA=0.10 µN and frequency f=100 Hz. Data from regions approximately 10×10 µm in size were collected using a scanning frequency of 0.2 Hz. The rest of the procedures was as in Toledano-Osorio *et al.* (2018). On the same surfaces, an atomic force microscope (AFM Nanoscope V, Digital Instruments, Veeco Metrology group, Santa Barbara, CA, USA) was employed for topography mappings. A 10 nm radius silicon nitride tip (Veeco) was attached to the end of an oscillating cantilever that came into intermittent contact with the surface at the lowest point of the oscillation. Changes in vertical position of the AFM tip at resonance frequencies near 330 kHz provided the height of the images registered as bright and dark regions. 10 x 10 µm digital images were recorded from each dentin surface, with a slow scan rate (0.1 Hz). For each image, 5 randomized boxes (1x1 µm) were created to examine the intertubular dentin (ID) and peritubular dentin (PD) nanoroughness at 24 h and 7 d of storage. Nanoroughness (SRa, in nanometers) and the collagen fibril diameter was determined as in Toledano *et al.*, 2018 (12). As the normality and homoscedasticity assumptions of the data were valid, numerical data were analyzed with ANOVA and Student-Newman-Keuls multiple comparison tests, with statistical significance preset at *p*<0.05.

- X-Ray Diffraction (XRD) and Transmission Electron Microscopy (TEM) analysis

Treated surfaces were submitted to XRD analysis. The X-ray micro-diffractometer (µXRD2) used in this study was a single crystal diffractometer with a 2-dimensional detector system Cmos Photon 100 (Bruker-D8 Venture, Wien, Austria), equipped with kappa geometry based goniometer 2D Detector and XRD 2D Scan software. The X-ray beam (Cu Kα line, λ = 1.5418 Å) was generated by a Cu Microforms source Iµs and generator settings of 50.00 kV/1.00 mA were employed. The sample to detector distance was 40.00 mm, 2Ɵ scanning angle range was from 10° to 80°. For TEM analysis, dentin samples were crushed into a fine-grained powder in a liquid nitrogen mortar and pestle, and 2% NaOCl was added to remove the surrounding collagen, with daily changes for a week (using a sonicator). Stack images were acquired, and the position of the stage was changed between -40 and +50° of α tilt, and collected every 10º. The samples were analyzed using a Transmission Electron Microscope Zeiss Libra 120 (Oberkochen, Germany) at 120 kV in bright-field (BF), and selected area electron diffraction (SAED) patterns were also captured. The rest of the procedure was as in Toledano *et al.*, 2018 (2).

## Results

- Nano-DMA analysis and Atomic Force Microscopy (AFM) analysis

**Figure 1:**
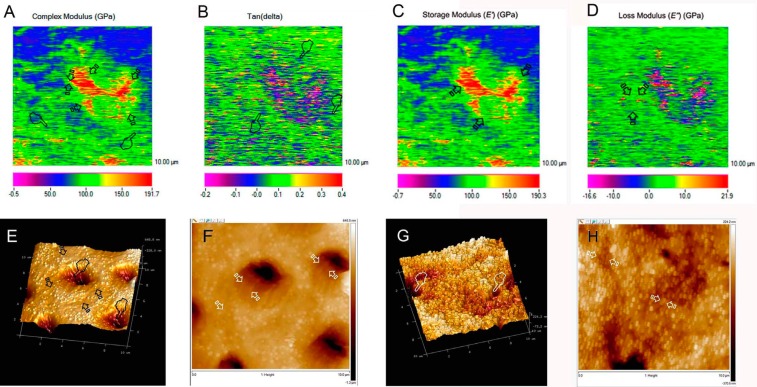
Scanning mode nano-DMA analysis of the map of the complex modulus (E*) at the cervical dentin treated with Ag-NPs, obtained at 7 d (A) time point. In the color scheme shown, the red color corresponds to the highest value of the locally measured moduli, likely corresponding to the highest resistance to deformation of the peritubular dentin (arrows). E* referred to intertubular dentin appears in bluish green (pointers). The pixel data array at the mapping are organized according to E* distribution that concurs with a clear delimitation between intertubular and peritubular dentin (faced arrows). Scanning mode nano-DMA analysis of the map of the tan *δ* at the cervical dentin treated with Ag-NPs, obtained at 7 d (B) time point. In the color scheme shown, the red color corresponds to the highest value of the locally tan *δ* value moduli, potentially associated to tan *δ* of intertubular dentin (arrows). The capacity for getting rid of the energy (tan *δ*) at peritubular dentin after 7 d of storage is represented by the blue-green diffused marks (pointers), at the mapping (B). Scanning mode nano-DMA analysis of the map of the storage modulus (*E’*) at the cervical dentin treated with Ag-NPs, obtained at 7 d (C) time point. In the color scheme shown, the red color corresponds to the highest value of the locally measured moduli, likely corresponding to the highest ability to stored energy of the peritubular dentin (arrows). *E’* referred to peritubular dentin appears in red yellowish. The pixel data array at the mapping are organized according to E* distribution that concurs with a clear delimitation between intertubular and peritubular dentin (arrows). Scanning mode nano-DMA analysis of the map of the loss modulus (*E’’*) at the cervical dentin treated with Ag-NPs, obtained at 7 d (D) time point. In the color scheme shown, the red color corresponds to the highest value of the locally (*E’’*) value moduli, potentially associated to loss modulus of peritubular dentin (arrows). The greatest ability of dentin to dissipate energy corresponded to peritubular dentin after 7 d of storage, and is represented by the dark-green areas (arrows), at the mapping. AFM 10 x 10 μm top-view and surface plot image of cervical dentin after applying Ag-NPs, at 24 h (E) of storage. Dentinal tubules remained visible and open (pointers). AFM heights (10 x 10 μm) (F) of this partially demineralized dentin surface with open dentinal tubules and very narrow fibrils width (faced arrows). AFM 10 x 10 μm top-view and surface plot image of cervical dentin after applying Ag-NPs, at 7 d (G) of storage, with open dentinal tubules. AFM heights (10 x 10 μm) (H) of this partially demineralized dentin surface. Dentin tubules were open (pointers) and thin collagen fibrils discovered (faced arrows). The dentin surface showed much more organized structure in contrast to the loose and randomly aligned fibril matrix of the untreated samples.

The nano-DMA properties of the cervical dentin surfaces at PD and ID were influenced by the type of NPs applied (*P*<0.05) and by the storage time (*P*<0.05). Interactions between factors were also significant (*P*<0.05). Mean and SD of complex modulus (E*), tan delta (*δ*), storage modulus (*E’*) and loss modulus (*E”*) are represented in  Table 1 and Figs. 1A-1D. Complex modulus (E*) assessed at 24 h of storage attained the lowest values at ID surfaces treated with undoped-NPs. At PD, undoped-NPs achieved the lowest E* at 24h of storage, and dentin treated with Ag-NPs attained the lowest E* among groups, at 7 d ( Table 1, Fig. 1A). After 7 d of storage, untreated dentin achieved the lowest *δ* at ID, and at PD Ag-NPs attained the highest tan *δ* values. Negligible data were presented at ID treated with Ag-NPs at 24 h, and at PD treated with undoped-NPs and Ag-Nps at 24 h of storage. An increase of tan *δ* in dentin treated with Ag-NPs was also observed at 24 h. Similar trend was followed at PD ( Table 1).

**Table 1:**
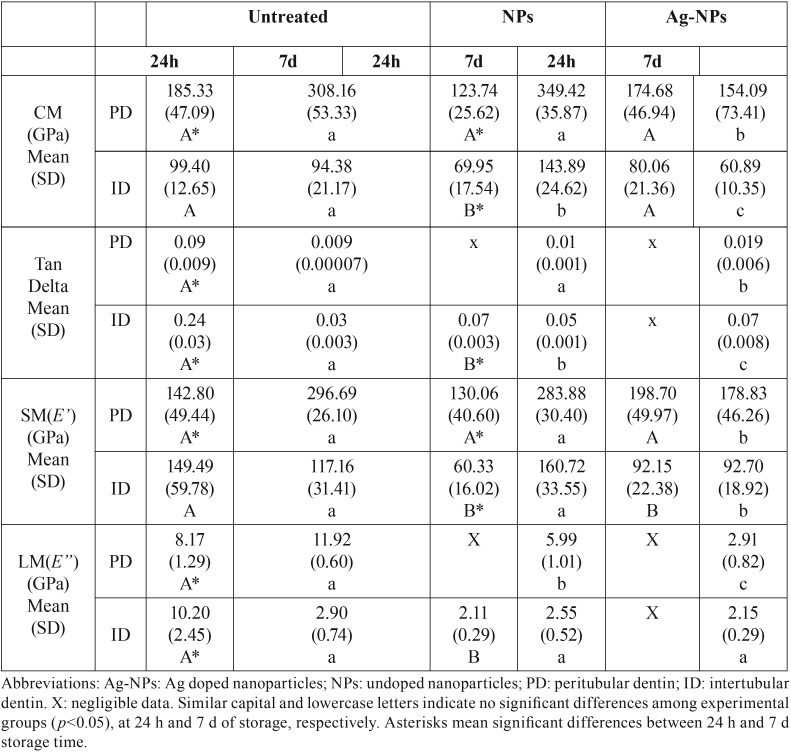
Mean and standard deviation (SD) of Complex Modulus (GPa), tan δ, storage (*E’*) and loss (*E’’*) modulus (GPa) attained for experimental dentin-treated surfaces.

Nanoroughness (SRa) of dentin surfaces was influenced by the type of NPs applied (*P*< 0.05) and by the storage time (*P*<0.05); interactions between factors were also significant (*P*<0.05). Mean and SD of nanoroughness are presented in  Table 2. The bandwidth of the collagen fibrils were also influenced by the type of NPs applied (*P*<0.05) and by the storage time (*P*< 0.05); interactions between factors were also significant (*P*<0.05). Mean and SD of the fibrils width are presented in  Table 2. At both 24 h and 7 d time points, dentin treated with undoped-NPs attained the greatest bandwidths among groups.

**Table 2:**
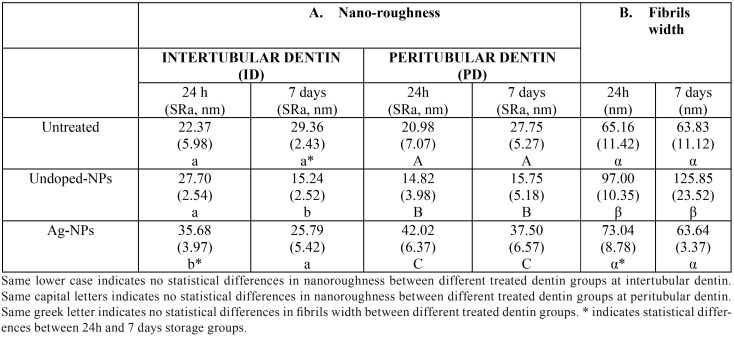
Mean and standard deviation (SD) of nano-roughness and fibrils width in untreated and undoped nanoparticles, Ag doped nanoparticles treated dentin.

-X-Ray Diffraction (XRD) and Transmission Electron Microscopy (TEM) analysis

**Figure 2:**
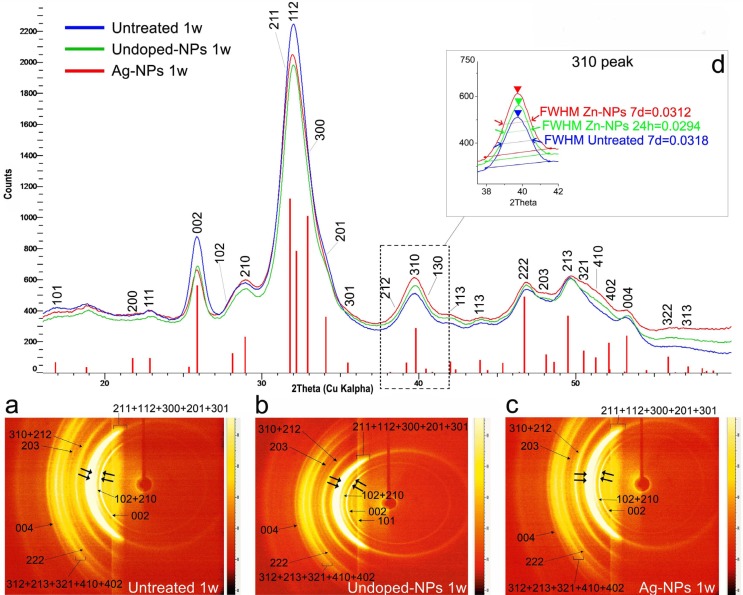
Refined µXRD2 profiles of untreated, undoped-NPs and Ag-NPs treated dentin after 7 d of PBS storage. The corresponding Debye-Scherrer rings are shown in inset a (untreated), b (undoped-NPs) and c (Ag-NPs). Double arrows mean strong diffraction rings. Vertical bars represent HAp peaks. Inset d represents a truncated µXRD2 profile after observing the reflection at 212, 310 and 130 peaks and further full width half maximum (FWHM) measurements after 7 d.

The analysis of µXRD2 profiles of cervical dentin showed that the physical broadening full width half maximum (FWHM) of peaks at 002 (2Ɵ, 25.900º; centroid peak position Ɵhkl, 0/0/-2; I, 10977386) reflection, after observing data plotted by the reduced full width and extended height at half maximum of the phosphate band, was higher (~1.1 fold) in untreated dentin surfaces when compared with the treated groups, after 7 d of PBS storage. When dentin was treated with undoped-NPs, after observing the reflection at 211 peak and the diffraction ring corresponding to 211 and 112 planes (Fig. 2, inset b), it may be noted higher crystallinity values than those obtained after assessing untreated (Fig. 2, inset a) and Ag-NPs treated dentin (Fig. 2, inset c). This was confirmed after observing the Table 3. A qualitative estimation of the size of the coherently scattering domain (i.e. the crystallite size) is reported in  Table 3. The shortest [τ002 (H)] (9.185 nm) and narrowest [τ310 (L)] (4.57nm) crystallite size, after 7 d of storage, corresponded to the untreated dentin ( Table 3). In the group of dentin treated with Ag-NPs, the dentin crystals attained the highest grain size [10.25 nm (τ002), 4.94 nm (τ310)] ( Table 3). Texture indices (R*hkl*) in dentin polycrystalline structures were calculated. At 002 plane, the texture assessed at the cervical dentin treated with all NPs solutions followed the trend: untreated dentin < Ag-NPs < undoped-NPs. At 310 plane, the trend was: Ag-NPs < undoped-NPs < untreated dentin ( Table 3).

**Table 3:**
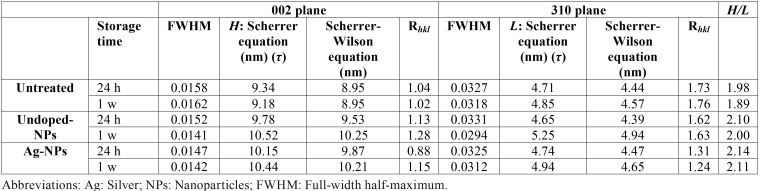
Micro-X-ray diffraction pattern analysis approach of dentin surfaces after nanoparticles application.

**Figure 3:**
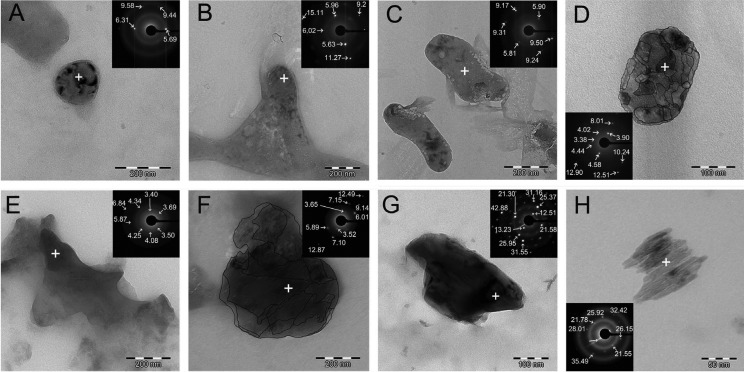
(A,B), Bright-field of an assembly of block-like or rounded apatite crystals of cervical dentin treated with Ag-NPs, after 24 h storage. At the images at B, it is observed that the particles have a domain of locally aligned crystal arrays, showing the staggered order of polygon crystallites (scale bar: 200 nm). (C,D), Bright-field of an assembly of rounded or hexagonal apatite crystals of cervical dentin treated with Ag-NPs, after 7 d storage. At the image D, it is observed that the particles have multiple domains of locally aligned crystal arrays with tendency to agglomerate. Profiles at D were highlighted to remark the overlapping [scale bar: 200 nm (C), 100 nm (D)]. Insets (top right), which correspond to a selected area electron diffraction (SAED) d-values of micron and submicron size (+), are also shown. Diffractography depicts diffuse halo rings and d spacing values of submicron size crystals, in general. The diffraction patterns show diffuse halo rings indicating the presence of amorphous structure. (E,F), Bright-field of an assembly of polyhedral and rounded/drop-like apatite crystals of untreated cervical dentin, after 24 h and 7 d storage, respectively. At the image B, it is observed that the particles have multiple domains of locally aligned crystal arrays with tendency to agglomerate. Profiles at B were highlighted to remark the overlapping. (scale bar: 200 nm, respectively). Diffraction pattern containing diffuse halo rings only, indicating some amorphous structure and clear d spacing values, meaning the presence of remnant crystallite matter. (G,H), Bright-field of an assembly of a polymorphic apatite crystals of cervical dentin treated with undoped-NPs based-gel, after 24 h and 7 d storage, respectively. The images show that the apatite crystal are made of overlapped order of the polycrystalline structure [scale bar: 100 nm (G), 50 nm (H)]. Images also exhibit that the particles have multiple domains. The labeled lattice planes of hydroxyapatite crystals, plate-like shaped, are clearly unveiled in H. Insets (top right), which correspond to a selected area electron diffraction (SAED) d-values of micron and submicron size (+), are also shown. Note that the spots are lightly delineated in these patterns, indicating polycrystalline structure. It also confirms the presence of hexagonal apatite.

After 7 d, the untreated cervical dentin exhibited, in bright-field (BF), distinct morphologies as rounded/drop-like crystals, polymorphic apatite crystals in strata when undoped-NPs were used and block-like or rounded apatite agglomerate crystallites when Ag-NPs were employed. They were transparent enough to observe their lattice, and also relatively stable enough for tilt series acquisition (Figs. 3A-3D). The polymorph/polyhedral crystals were confirmed to be more amorphous in nature than that organized in strata or in rounded morphologies (Fig. 3F), as observed from the diffuse ring pattern (insets at Figs. 3E, 3F).

## Discussion

This study has shown for the first time that the strong interaction of Ag-NPs with HAp structure provoked poor maturation and scarce functional remineralization with ultrafine nano-crystallites at cervical dentin; these hexagonal nanoplate crystallites, with scarce crystallinity, had preferred orientation in c-direction with tendency to agglomerate toward random orientation.

Considering that both the storage modulus (*E’*) and the loss modulus (*E’’*) (Figs. 1C, 1D) ( Table 1) are involved in the viscoelastic expression of the complex modulus (E*) and tan *δ*, only E* and tan *δ* will be discussed. The complex modulus (E*) is a measure of the resistance of a material to dynamic deformation (13). After 7 d of storage, intertubular untreated dentin and dentin treated with Ag-NPs attained similar E*, but at peritubular dentin the lowest resistance to deformation was obtained in dentin treated with Ag-NPs ( Table 1), (Fig. 1A), denoting poor functional or intrafibrillar remineralization (14). This indicates a decrease in apatite maturity and in dentin mechanical properties (15). The moderate modulus that was obtained complies with scarce peritubular mineralization and generalized open tubules (Fig. 1G).

This scarce remineralization was confirmed by nanoroughness measurements, as dentin treated with Ag-NPs attained the highest SRa values ( Table 2). An increase in roughness is associated to poor mineral maturation and it is a sight of scarce intrafibrillar remineralization. Accordingly and contrary to the dentin treated with undoped-NPs, the fibrils width did not change when compared with the untreated group, confirming the absence of mineral gain at fibrils (Figs. 1F, 1H) ( Table 2). At both PD and ID treated with Ag-NPs, after 7 d of storage, no mineral deposits (Fig. 1G) and the highest tan *δ* values were observed ( Table 1) (Fig. 1B). The greater tan δ, the lower proportion of energy available in the system for recoil and/or failure (16). Dentin treated with undoped-NPs attained both remineralization of dentin and mineral precipitation within the dentinal tubules (data not shown), but the maxima discrepant tan δ values (~5 fold) when ID and PD were compared, after 7 d storage. In the present study, discrepancy of tan *δ* values between ID and PD became potentially associated to zones and signs of energy concentration as bridge-like structures, stick-slip images, indicating sights of energy dissipation (17). Signs of energy concentration hardly appeared at the dentin surface treated with Ag-NPs (Fig. 1G), due to the relative higher homogeneity of viscoelastic properties.

When µXRD2 of dentin treated with Ag-NPs was analyzed, a FWHM decrease, nearly one order of magnitude, was observable when data from specimens stored 7 d were compared with the untreated samples at both 002 and 310 planes ( Table 3) (Fig. 2). These sharper and better resolved peaks or peak broadening (3) parallel to the *c*-axes, indicated less amorphous HAp than that observed at the untreated group. Diffractography patterns gradually transform from broad diffuse peaks at cervical dentin treated with undoped-NPs or Ag-NPs at 24 h, to sharper and more crystalline peaks after 7 d ( Table 3) (Fig. 2). This confirms that amorphous phase itself is dynamic in nature. The SAED pattern observed in undoped-NPs (inset at Fig. 3H) revealed polycrystalline diffraction rings with interplanar spacing that was consistent with that of HAp (18).

The diffuse ring observed at SAED (Fig. 3D, inset) when dentin was treated with Ag-NPs indicated a decrease in crystallinity (19), probably amorphous calcium phosphate or octacalcium phosphate, contained within the apatite plates. This amorphous mineral can provide many active sites to attract Ag+ ions, promoting the deposition of Ag-NPs on the mineral substrate (18). Nevertheless from analyzing the inset at figure 3D, it can be inferred the presence of other crystalline species such as HAp, silver sulfide (7), or even silver phosphate phase (20). After 7 d, mean crystallite size along a direction parallel and perpendicular to the c-axis [τ002 (H)] and [τ310 (L)] of the dentin treated with Ag-NPs was ~1.1 and 1 folds higher respectively than those of untreated dentin. In the HAp crystals space structure, the c-plane contained more negatively charged group such as PO43- ions and OH- ions, while the a-plane had more positively charged Ca2+ ions and consequently, more Ag-NPs may be attached at the c-plane (18). Apart from that, H/L ratio increased in dentin treated with Ag-NPs more than in the rest of the groups ( Table 3). This finding suggests a strong interaction of Ag-NPs with the HAp structure (21).

Texture is the distribution of crystallographic orientation of a polycrystalline sample. It accounts for changes in microstructure, proving a great influence on materials properties, as cracking resistance (22). For R≈1, the grains were considered randomly oriented (23). The closest group to this criterion after 7 d of storage was the Ag-NPs, (R*hkl*= 1.24) ( Table 3). After Ag-NPs treatment and 7 d storage, hexagonal nanoplate arrays, with regular and clear contour with tendency to agglomerate, were created, with a length reaching of ~200 nm (Fig. 3D) and crystallite sizes of 10.44 nm x 4.94 nm ( Table 3). It is speculated that this morphology may be due to the effect of surrounding silver phosphate phase, Ag3PO4 (20). The predominant ring pattern corresponding to figures of two semi-circles instead of a continuous ring, when Ag-NPs were 7 d applied (Fig. 3D), show that the crystallites have preferred orientation in c-direction (2). These results indicate that Ag-NPs induced changes in the structure/chemistry relationship that perform in synergistic manner. Therefore, the null hypothesis that was formulated in the present study must be rejected. There are several potential limitations of this study. Firstly, we measured dynamic rather than static nanomechanical properties. This new approach will throw light on whether Ag-NPs provokes definitive functional remineralization or not at cervical dentin, by measuring nanohardness and Young’s modulus. Secondly, the extended time period of 7 d for this study was short. Within the limits of this study, it may be concluded that Ag-NPs had negative effect on the mechanical properties of cervical dentin, though the energy dissipation did not damage the dentin structure. Remineralization at dentin was not produced after Ag-NPs application, though improved crystallinity may lead to increase stability of the apatite that was generated at the dentin surface. The present study, based on a new type of Ag-NPs, suggests a new class of nanomaterials for dental applications, with incorporated bactericidal effect.
